# Supplementary material for: Ancient origin of lubricated joints in bony vertebrates
Source: eLife. 2016 Jul 19;5:e16415. doi: 10.7554/eLife.16415 (PMC4951194; doi:10.7554/eLife.16415)
Supplement: Supplementary file 1. — (1A) Forward and reverse primers used to amplify zebrafish cDNA for in situ hybridization probe generation. (1B) Sequences used to generate in situ hybridization probes for stickleback and spotted gar prg4 genes. (1C) Forward and reverse primers used to genotype prg4a and prg4b mutant zebrafish. DOI: http://dx.doi.org/10.7554/eLife.16415.014 [file elife-16415-supp1.docx]

Supplemental file 1A.

In situ hybridization probes for zebrafish genes

| **Probe** | **Primer sequence/Template** | **Fragment size (bp)** |
| --- | --- | --- |
| *prg4b* | Fwd: CAGCATGAAAATGGAAAGAGA  Rev: GCAGCTCTCAGCCTCCTAAA | 661 |
| *prg4a* | Fwd: GAACGTCAAGATTAGCGGTGA  Rev: GCTTTTCTGAGTTTTAATGAAGCA | 508 |
| *has3* | Fwd: GCATTTCTATCCCTCCACCTTCTC  Rev: GGGTCTGCTGATTGAGCCAACGC | 919 |
| *acana* | Fwd: TGTGAACCAAACCCTTGTGGAGC  Rev: CGAAGGCTTGCTCCTCTGGAG | 367 |
| *col10a1* | Fwd: GTCTAAAAGGTGACAGAGGAGTACCT  Rev: GTAGACACTGATCAGTAACAAGGAAACA | 1421 |
| *matn1* | Fwd: CTCAGTTTCCTGGTGGACAACAG  Rev: TGGATAATCAAGCATTCCCGC | 834 |

Supplemental file 1B.

DNA sequences for in situ probe generation via gBlock Gene Fragment synthesis.

| **Species** | **Gene** | **Sequence** |
| --- | --- | --- |
| three-spined stickleback | *prg4b (ENSGACG00000007505)* | TCAGACAAGCTGTAAAAGTCGATGTGGTGCTGAGTACTACAGGGGCTACATGTGTCAGTGTGACTACAACTGCCTGTCTTATGGAGAGTGCTGCAAAGACTTTGAATCCCAATGCACCACAAAAAACTCATGCAGAGGACGGTGTGGAGAAGACTTCAAAAGAGGCCGGCTGTGTACCTGCGACCCTAAATGCAATGATTACAAACAGTGTTGTCCAGATCACAAAACCCACTGTGACGCAGAAGATATCAACGGAGAAATTCTTCCCAATGAGGATTCTTCCAATAACGAAGTGGGAGACCCAGAAGAAATTCCATTCCCAGAAAGCACCAGTGACCCAGCTGACCTGCTGGATCCCATTCCTACCGAACCCATCGAGGATCCAGATACTCTGGAGGTCCCTACAGAGACATTTACAGCCTCCTCCCAGGCACAAACGACTGTCTCAGACAAAGAACCAACACAGGCCGAGGACGGCTCCGCCTCACCGTCCCCCACCGCTGTAGAAGCCTCAACTGAGAGCACAGCCAAACCCACGGCGGTCGAGATGGCGGGGCAGGAAGAAGTACTCCCCGAGGACGACAATCCTGCCGAGGTTCTGACTGAAGCCCCCTCTGATGAGCCAGAGGAAACATCTCTCTCCAAAACAACTGTGGCCCCAATATCCACAGAACCCACACAGGCCTCCGATGGCCCGGACGACTCTCAGGTCACCACCCTCTCTGCCTCAATGCCGGACCCGACACCCAT |
| three-spined stickleback | *prg4b (ENSGACG00000007501)* | ATGGTTCGATCAGCAGCATCTCATCTTGAGCCAGCAGTTAACATCCGGACAGCTTGGAGAGGTTTCCCCTCCACCATCACAGCTGCTGTGTCCGTCCCGAGCAGAAGAGATCCAGAGGGATACAAATACTACGTCCTATCAAGATCCACATCCTACAATGTGAGGATTTCTAGCAATCGTCCCATCGTTCCTGCTCCTGCAACCAACGCGCCACCACAGAGCAACGACTTCTTGAAATGCCCAAAGAGAGCGTGAGGAAGGAAAGAGGCATCAAATGCGGCTCATCTTACAGCTCTCAAGTTTATTACATACTTCATAAAACACAACGCTTAAGCATGGACTGATAGCAAAAATGTACTTTAACAGTATATAAAGAAAAAGAAAACTTAAAAACAACAAGCTTCAAAACACGCAAACATTGTCATGGCTGAAAAGAGAATATGAAAACATCGGTTTAAAAATATCTAATGTGTTTTTGAAGGGAATGGAAACCTTTTCTGTCACTGAGTTTTCTCACTGCTGCTTCATCTCACGAATCGTCTCCCATGAACACCCCGAGAAGGAAATCCCTGACCTGTTGGGAGATTTAAAAAAAAAAAGTCAACCTGCTATAGTGGCGTAAAGAATTTGTACAGTGGCTGTATAACATGTGTAAGAAATGTAAA |
| three-spined stickleback | *prg4a* | TCGCCGCTGGCTCAGGAGTCGCCAACCGCAGACCCAGCACCCTGCAGGACGTCGCCCAGGCCTCGGGTCTACTGGACGGGGGTCTACTGGAGCTGGGGACAGGTGGAGTCCTTAATGATGTCGACCTGTGCAGTGATTCTCCCATCAATGGACTCACAGCTCTTAGCAATGGGACCATTCTGATATTTAAAGGGGACGTGTTCTGGTCAGTGGACGGCGTCAGTCGCTCAGCGGGACGTCCCCAGAGCATCATGGACACTCTGGGCGTCCCCTCTCCCATCGACACCGTGTTCACGCGCTGCAACTGCCTCGGACACACCTACATCATCAAGGGAGACCAGTACTGGCGCCTGGATGGGGACATGGTGATGGAGCCGGGTTACCCCAAACCTCTGGTCTCAGAGTTCCCGGGTCTGACAGGAAGCATCAGCGCCGCACTGGCAGCCCCAGCCGCCGGCGGCAGAGCGGAGACAGTGTACTTCTTCAAGAGCGGAGACATCATGCAGAGCTTCACCTTCCCAGCAGCCAGCACTCCCTCCTGCAGCAAGACTCCCAGCAAGACTGCCAGCGGATCCGCGAGGGGCCGCTCGGCTCGGCAGGCGGAAGTTCTTCTCAGTGGGGAGATCAACGTCAGAGTGTCTCTGGCCGGATTCCCCTGCCCGGTCACGTCGGCCCTGTCCGTGCCCAGTCCTCGGAGGAGAGACCCATACGAGCACTACGTCTTCTCTGGAC |
| spotted gar | *prg4* | GTTCCCAGCACTTCGTCAGGAACTGTCACAATCCAAAGCACCGTTCCCTCAAAACATCCCAGTGACTCAGTAGGTGAAAATAATGATACCAACATCTGCAGCGGCCGTCCAGCTAATGCACTGACGACTCTACAAAATGGAACAATGATTGTTTTCCGGGGACATTTTTTCTGGACATTCGGCTCCAACGGAGCTCTAGGACACCCTCGCAGGATAACAGAAGAGTGGGGAATTCCTTCTCCCATAGATACGGTCTTCACTCGCTGTAACTGCCAAGGGAAAACTTTCATTTTCAAGGCAAACGAATACTGGAGATTTGACAATGGGGTGATGGACAGGGGGTACCCCAAACTGATCTCCAAAGGGTTTGGAGGAATGTCAGGACGAATAACTGCTGCTCTGTCTGTGCCTGCTTTTGGAAAACGAAGGGAAGCCGTTTATTTCTTCAAACGAGGAGGCTTGGTGCAAAAATATTCCTACAAACAAGAACCTTCCCAGTCTTGTGGCAAACCCCAAACCCAGTACCGCATCTACAGCCTCCACAGCAGGATAACTAGACAAGCAGGTCCAGTATATCTTGGAAAAGAAATCAACATTAAACTGACATGGAAAGGTTTTCCTACATCAGTTACTTCAGCTATATCTCTACCCAACTCCAAGAAACCAGATGGATATGATTATTTTGTATTTTCAAGGGCCAAGTACTACAACATTATAATTAACAGCGACCAGCCAGCTGTGACTCCTCTG |

Supplemental file 1C.

Primers used for genotyping *prg4a* and *prg4b* mutants.

| **Primer Name** | **Sequence** | **Amplicon size in WT** | **Amplicon size in Mut.** |
| --- | --- | --- | --- |
| prg4a_null_F | TGTGGGCTGATACTGTTTTGGG | NA | 431 |
| prg4a_null_R | GGGTAGTGAGTGAGGATTTGTGGC |  |  |
| prg4a_exon4_F | CAGCCTCTCATCATTTTCTTACCC | 335 | NA |
| prg4a_exon4_R | GTCATCATACCGCAGTGTGTGC |  |  |
| prg4b_null_F | GGACGGAGAGGGGAACAC | NA | 320 |
| prg4b_null_R | AAGCTCCTCCCTAGTTTTGCTAAATG |  |  |
| prg4b_exon7_F | TCCCAAGAAAGACACCTGCTCC | 394 | NA |
| prg4b_exon8_R | GCCATTTGAACTGACCTCTGAACAC |  |  |
